# Protocol for renal artery embolization via caudal artery in rats

**DOI:** 10.1016/j.xpro.2025.104080

**Published:** 2025-09-11

**Authors:** Yi Jiang, Peng-tao Chen, Chao-yi Qian, Chen-hang Sun, Wei-jun Wang, Hui-yan Ding, Yi-nan Ding

**Affiliations:** 1 Department of Interventional Radiology, Zhejiang Cancer Hospital, Hangzhou Institute of Medicine (HIM), Chinese Academy of Sciences, Zhejiang Key Laboratory of Imaging and Interventional Medicine, Hangzhou, Zhejiang 310022, China; 2 Department of Radiology, Zhejiang Cancer Hospital, Hangzhou, Zhejiang 310022, China

**Keywords:** Health Sciences, Model Organisms, Material sciences

## Abstract

Transvascular embolic materials are vital for acute hemorrhages and tumors, but newly developed materials require preclinical validation. Here, we present a protocol for renal artery embolization through the caudal artery of rats. We describe steps for rat anesthesia, heparinization of surgical materials, and isolation of the rat caudal artery. We then detail procedures for puncturing and intubating the rat caudal artery and superselective renal artery embolization.

## Highlights

• Steps for rat anesthesia and heparinization of surgical tools
• Procedures to isolate the rat caudal artery via dissection
• Instructions for caudal artery puncture and catheter insertion
• Guidance on DSA-guided renal artery embolization with microspheres

## Abstract (editor highlights)

Publisher’s note: Undertaking any experimental protocol requires adherence to local institutional guidelines for laboratory safety and ethics.

## Graphical abstract

**Figure:**
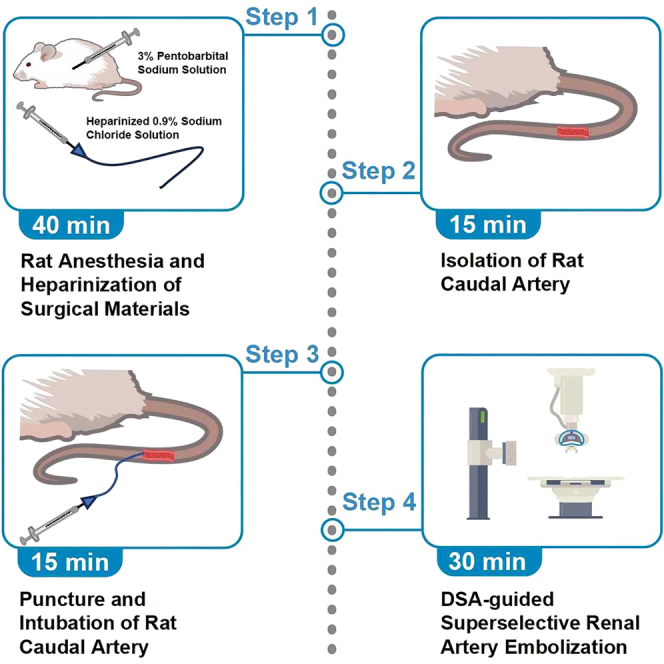

## Before you begin

Since Dr. Seldinger originally developed the percutaneous vascular puncture technique, transvascular interventional therapy has made significant advancements in clinical medicine due to its less invasive and efficient benefits.^1^ This approach has been extensively utilized across various clinical disciplines, including cardiology, cancer, and emergency hemorrhage.^2^^,^^3^^,^^4^ By accurately administering embolic agents into blood vessels, one can obstruct the blood supply to malignancies for localized ablation and swiftly occlude ruptured vessels to attain hemostasis. Nevertheless, the design constraints of transvascular interventional consumables render it challenging to utilize mice, frequently employed in fundamental research, due to their narrow blood arteries. Consequently, rats of moderate size and appropriate vascular conditions have increasingly emerged as the predominant animal models in transvascular interventional research, serving as an optimal surrogate assessing the efficacy of novel embolic materials and investigating embolization mechanisms.

Currently, traditional vascular interventions in both human and animal models are conducted via femoral artery puncture. This method entails a laborious operational procedure in animal models (necessitating skin preparation, incision, and complete exposure of the femoral artery), inflicting considerable damage on the animals, has a substantial risk of complications, and poses significant challenges for repeated intubation. Consequently, caudal artery puncture and intubation represent a practical and secure procedural technique for rats.

Wang et al. had presented a technique for caudal artery puncture in rats in their published publication.^5^ Nevertheless, the rats utilized in the study weighed around 700 g, although the standard weight for adult rats generally ranges from 250 to 300 g. Many researchers encounter challenges in acquiring rats that exceed 400 g in weight. The caudal artery of a 300-g rat is clearly narrower than that of a 700-g rat, complicating puncture procedures. This protocol aims to furnish researchers with a more standardized technique for caudal artery puncture. The essential interventional instruments and consumables—comprising micro-guidewires, 20G indwelling catheter, 26G indwelling catheter, and micro-catheters—are widely accessible in the marketplace. This allows our method to function as a replicable methodological reference for others in the same research area.

### Innovation

This protocol innovates by optimizing rat caudal artery embolization for standard 250–300 g rats, addressing limitations of previous methods using 700 g rats. It employs a sequential catheter strategy: a 26G indwelling catheter for initial access, followed by a 20G catheter to accommodate a 1.6F microcatheter, enabling precise navigation in narrower vessels. The workflow integrates DSA-guided superselective renal artery targeting with standardized heparinization solutions (2.5 U/mL for consumables, 6.25 U/mL in vivo) to reduce thrombosis risks. Unlike traditional femoral artery puncture, this minimally invasive caudal approach minimizes trauma, lowers complication rates, and facilitates repeated procedures. By using widely available instruments and clear troubleshooting for challenges like arterial spasm or catheter sticking, the protocol enhances reproducibility, making it a practical advance for preclinical validation of embolic materials.

### Institutional permissions

All animal experiments involved in this study were strictly conducted in accordance with relevant ethical guidelines and have obtained approval from the “Ethics Committee of Zhejiang Cancer Hospital” (Approval No.: 2025-04-023). All procedures were performed in compliance with the standards of the *Regulations for the Administration of Experimental Animals*, minimizing animal suffering and ensuring their welfare to the greatest extent.

### Preparation of consumables and instruments

**Timing: 20 min**

Before surgery, it is essential to prepare surgical instruments and consumables.

1. Cover the instrument cart with a disposable surgical drape, systematically arrange the essential equipment for the procedure.
2. Ensure that surgical instruments are sterilized by high-pressure steam (e.g., forceps, hemostatic forceps, needle holders, surgical scissors, stitch scissors, micro scissors, surgical handles, surgical blades, glass dissector etc.) are prepared for use.

***Note:*** The consumables essential for vascular intervention include micro-guidewires, micro-catheter, 26G indwelling catheter, 20G indwelling catheter (without needle), and an appropriate amount of iodine contrast medium (iopromide), so as to ensure successful vascular puncture and intubation.

***Note:*** A 26G indwelling catheter is preferred for its smaller outer diameter, which facilitates vascular access and enhances procedural success. The lumen of a 20G Indwelling Catheter accommodates a 1.6F microcatheter and slightly exceeds the minimal inner diameter of rat tail arteries. For Sprague-Dawley rats weighing 250 ± 50 g, a 20G Indwelling Catheter is recommended, with adjustments to larger gauges permissible based on individual animal size.

**Figure 1:**
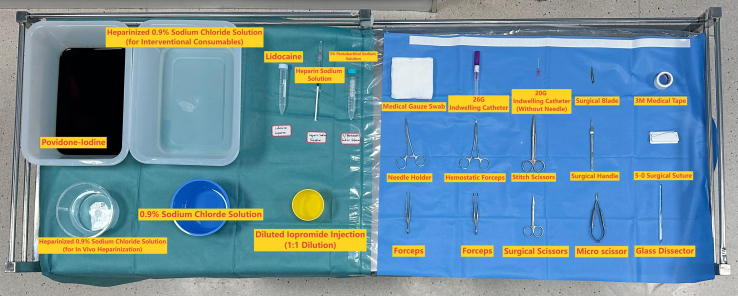
Partial surgical instruments display

3. The indispensable consumables and auxiliary supplies for surgery include iodophor for disinfection, heparin sodium for preventing intraoperative thrombosis, lidocaine as local anesthetic, 3% pentobarbital sodium as general anesthetic, 0.9% sodium chloride solution, 1 mL syringe, 5–0 surgical sutures for suturing and 3M medical tape (Figure 1).

***Note:*** Aseptic operation is of vital importance. The operating room should also be disinfected before surgery, and disposable consumables shall be used only once and must not be re-disinfected or reused.

## Key resources table

**Table:** 

REAGENT or RESOURCE	SOURCE	IDENTIFIER
**Reagent**		

Povidone iodine solution, 5%, 500 mL	Hangzhou Minsheng	N/A
Sodium chloride solution, 1,000 mL: 9 g	Sichuan Kelun	N/A
Lidocaine hydrochloride injection, 10 mL: 0.5 g	Harbin Sanma	N/A
Heparin sodium injection, 2 mL: 1,2500 units	Chengdu Haitong	N/A
Iopromide, 100 mL: 76.89 g	Bayer	N/A
Pentobarbital sodium, 10 g	Hangzhou Dawen bio	N/A
Hydroxyapatite microspheres, 50 μm, 1 g	Nanjing Junzhuo	N/A

**Biological samples**		

Sprague-Dawley rat, 250g–300g, male	Beijing Vital River Laboratory Animal Technology	N/A

**Others**		

1 mL Syringe	BD	300841
Medical gauze swab, 10 cm∗10 cm-8p	Zhende Medical	N/A
Medical tape	3M	N/A
5–0 Surgical suture	Jinhuan Medical	F503
26G Indwelling catheter	N/A	N/A
20G Indwelling catheter	N/A	N/A
Micro-Guidewires, 0.014-inch	Boston Scientific	N/A
Microcatheter, 1.6F	Cardiolink	N/A
Digital subtraction angiography (DSA)	Philips	UNIQ FD20
High-pressure syringe	Bayer	MARK5

## Materials and equipment

**Table:** Heparinized 0.9% sodium chloride solution (for interventional consumables)

Reagent	Stock concentration	Final concentration	Amount
Heparin Sodium Injection	12500 U/2 mL	2.5 U/mL	200 μL
Sodium Chloride Solution	9 g/L	8.9964 g/L	499.8 mL
**Total**	**N/A**	**N/A**	**500 mL**

**Table:** Heparinized 0.9% sodium chloride solution (for *in vivo* heparinization)

Reagent	Stock concentration	Final concentration	Amount
Heparin Sodium Injection	12500 U/2 mL	6.25 U/mL	200 μL
Sodium Chloride Solution	9 g/L	8.991 g/L	199.8 mL
**Total**	**N/A**	**N/A**	200 mL

***Note:*** All reagents should be stored at 4°C.

***Note:*** The maximum storage time is 2 weeks.

## Step-by-step method details

### Rat anesthesia and heparinization of surgical materials

**Timing: 40 min**

This step mainly includes preparatory work such as anesthesia of rats and heparinization of surgical materials.

**Figure 2:**
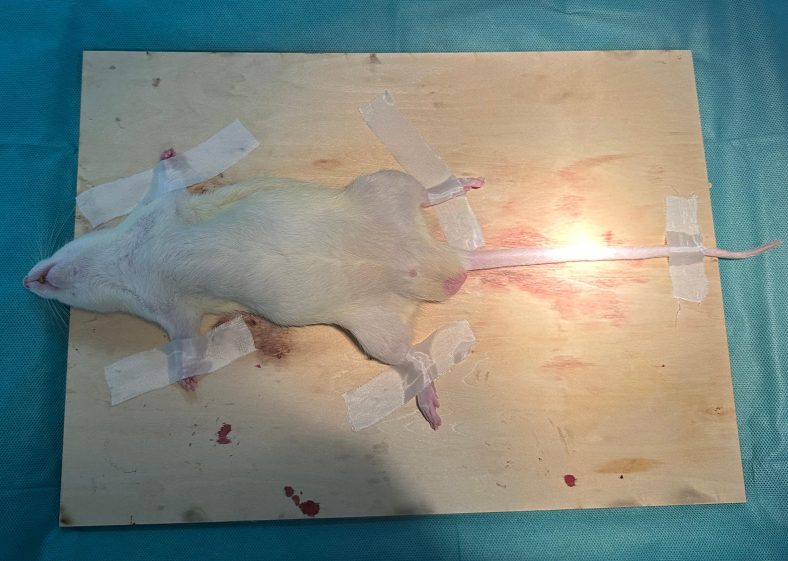
Place the rat on the operating table

1. Select healthy rats from the enclosure.
2. Firmly hold the nape of the rat’s neck with hand, invert it onto its back, and immobilize its hind legs to the experimental table.
3. Disinfect the lower abdomen using povidone-iodine. Insert a needle adjacent to the linea alba, pierce the dermis, and gradually move into the abdominal cavity.
4. Aspirate the syringe to verify that there is no blood, intestinal fluid, or urine. Gradually administer approximately 300 μL of a 3% pentobarbital sodium solution.
5. Monitor the rat’s respiration for fluidity. Pinch the hind paw; lack of withdrawal response signifies sufficient anesthesia.
6. After the anesthesia, arrange the rat supinely and secure its four limbs to the operating table (Figure 2).

***Note:*** The angle of needle entry for intraperitoneal injection should be moderate to avoid the risk of intestinal perforation or subcutaneous injection. The advised angle is between 30° and 60°, with 45° being optimal. The insertion depth must not exceed 1 cm.

***Note:*** The optimal anesthetic dosage of pentobarbital sodium for rats is 30–50 mg/kg. Please adjust the administration volume according to the actual body weight of the rats.

***Note:*** Rats usually regain consciousness approximately 2 h after being anesthetized, and this duration is longer than the total length of the experiment.

7. Soak the micro-guidewire in heparinized 0.9% sodium chloride solution (for interventional consumables) for 10 min (See materials and equipment setup).
8. Flush the microcatheter and indwelling catheter three times with heparinized 0.9% sodium chloride solution (for interventional consumables) using a 1 mL syringe.

***Note:*** Heparinized 0.9% sodium chloride solution is used in vascular interventional procedures to prevent catheter occlusion and thrombus formation.

***Note:*** The volume for each flush is approximately 3 mL.

### Isolation of the rat caudal artery

**Timing: 15 min**

This step primarily involves dissecting and isolating the caudal artery of the rat.

***Note:*** The surgeon should wear a surgical gown, sterile gloves, mask and cap, and maintain aseptic operation throughout the surgery.

**Figure 3:**
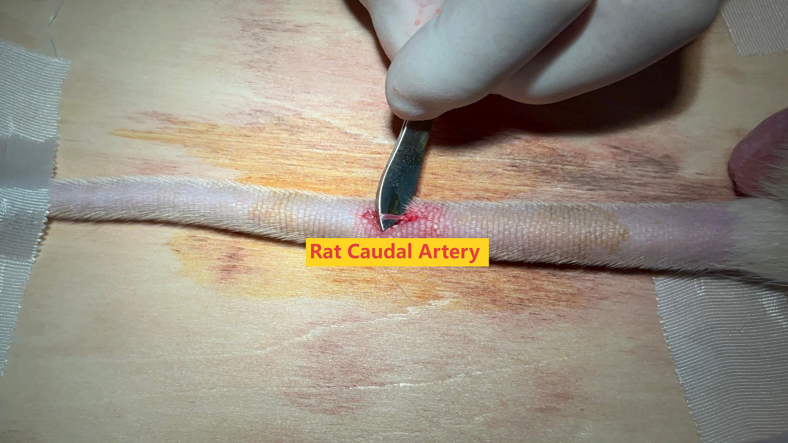
The isolated caudal artery of the rat

9. Adjust the height of the operating table to an appropriate position according to the surgeon’s needs.
10. Align and straighten the rat’s caudal artery, identifying the surgical site at the mid-upper portion of the caudal artery midline.
11. Focus the shadowless lamp on the surgical site.
12. Thoroughly disinfect the surgical site with povidone iodine solution.
13. Administer lidocaine subcutaneously at the surgery location for local anesthetic (Methods Video S1).
14. Make a 1–2 cm longitudinal incision in the surgical site with a scalpel, expand the incision and drop a small amount of lidocaine (problem 1-troubleshooting).
15. Expose and systematically dissect the fascia, along with the associated blood vessels and nerves, while isolating the caudal artery with glass dissector.
16. Stabilize the caudal artery using the tail end of the forceps (Figure 3, Methods Video S2).

***Note:*** As the fascia may obstruct the view during layer-by-layer dissection, partially transect the fascia when necessary to clear the field of vision.

***Note:*** Minor venous hemorrhage might occur during the isolation of the caudal artery due to inevitable vascular damage. Initially, ascertain if the caudal artery damaged, then implement temporary compression to manage the hemorrhage.

Methods Video S1. Disinfection and local anesthesia of the rat caudal artery, related to step 13

Methods Video S2. The isolation process of the rat caudal artery, related to step 16

### Puncture and intubation of the rat caudal artery

**Timing: 15 min**

This step primarily includes the procedures for puncture and cannulation of the rat caudal artery.

17. Elevate the caudal artery to an angle of roughly 30° using the tail end of the forceps (problem 2-troubleshooting).
18. Insert 26G indwelling catheter parallel to the caudal artery to a length of approximately 0.5 cm.
19. Carefully extract the stylet.
20. Insert the micro-guidewire into the 20G Indwelling Catheter and secure the tip of the micro-guidewire.

***Note:*** Before inserting the micro-guidewire, moisten a medical gauze with heparinized 0.9% sodium chloride solution (for interventional consumables) and apply the solution to the surface of the micro-guidewire.

21. Insert the 20G Indwelling Catheter into the end of the 26G indwelling catheter, directing the micro-guidewire to traverse the 26G indwelling catheter and access the caudal artery.

***Note:*** Due to the softness and frequent bending of the microcatheter tip, stabilize it using the indwelling catheter or alternative techniques to aid the micro-guidewire’s insertion into the caudal artery.

**Figure 4:**
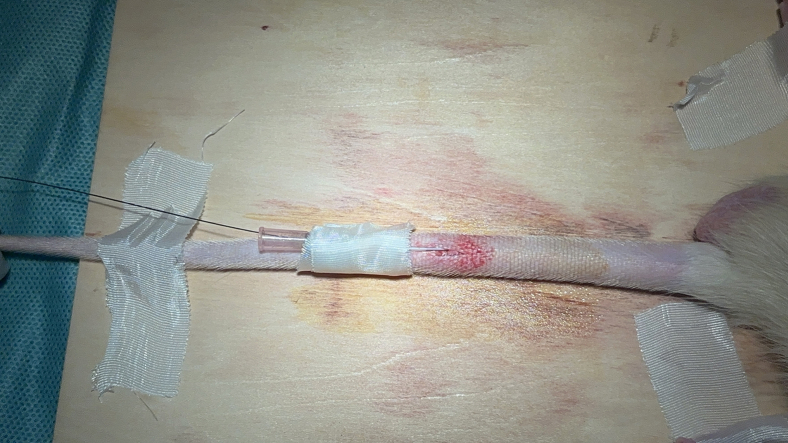
Successful puncture and fixation of the rat caudal artery

22. Once the micro-guidewire has entered the caudal artery, advance it a further 5–10 cm.
23. Remove both the 20G and 26G indwelling catheter.
24. Re-thread and insert the 20G indwelling catheter into the rat caudal artery (Methods Video S3).
25. Affix the 20G indwelling catheter to the tail with medical tape (Figure 4, Methods Video S4).
26. Advance a 1.6F microcatheter over the distal end of the micro-guidewire into the caudal artery. (Methods Video S5).

Methods Video S3. Puncture and indwelling catheterization of the rat caudal artery, related to step 24

Methods Video S4. Fixation of the catheter sheath after completing indwelling catheterization, related to step 25

Methods Video S5. The microcatheter advances into the caudal artery over a micro-guidewire, related to step 26

### DSA-guided superselective renal artery embolization

**Timing: 30 min**

This step mainly involves using a microcatheter to perform superselective catheterization of the renal artery and inject embolic microspheres.

27. Under DSA guidance, advance the micro-guidewire and microcatheter into the abdominal aorta (problem 3-troubleshooting).

***Note:*** DSA is set to pediatric mode, with a contrast acquisition frame rate of 6 frames per second.

28. Withdraw the micro-guidewire, then inject 1 mL of heparinized 0.9% sodium chloride solution (for in vivo heparinization) through the microcatheter (See materials and equipment setup).
29. Re-insert the guidewire and locate the orifice of the right renal artery under DSA.
30. After the micro-guidewire enters the right renal artery, guide the microcatheter into the right renal artery.
31. Withdraw the micro-guidewire and inject 1 mL of 1:1 diluted Iopromide to observe whether the right renal artery is opacified (problem 4-troubleshooting, Methods Video S6).

***Note:*** The diluent for iopromide is 0.9% sodium chloride solution.

32. Once the right kidney is confirmed to be opacified, connect the microcatheter to high-pressure syringe.

**Figure 5:**
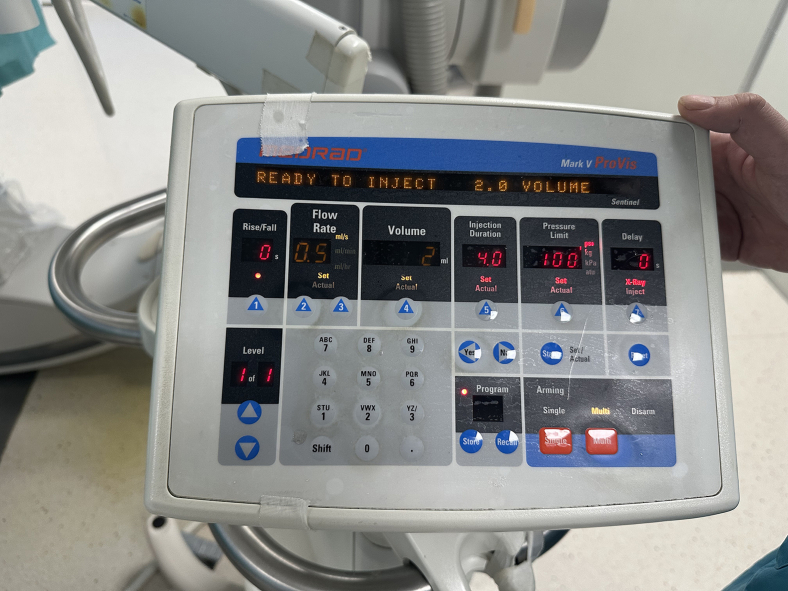
Real-time parameters of the high-pressure syringe

***Note:*** The parameters of the power injector are as shown in Figure 5.

**Figure 6:**
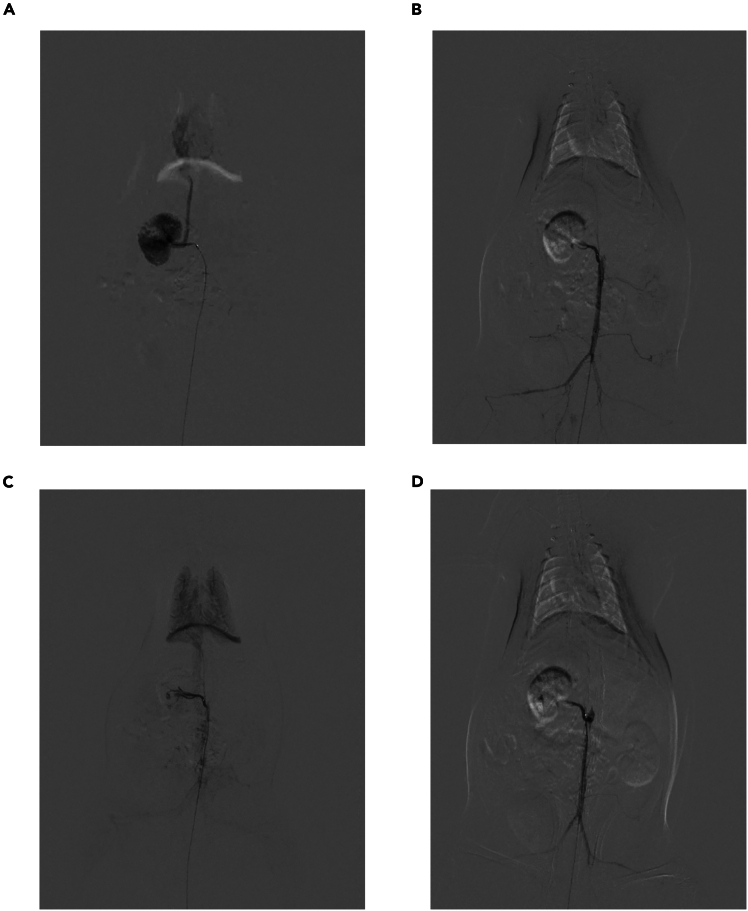
Microsphere embolization of angiography images before and after (A) is the angiography image before microsphere embolization; (B) is the angiography image after the first microsphere embolization; (C) is the angiography image 10 min after the first embolization is completed; (D) is the image after the second microsphere supplementary embolization.

33. The high-pressure syringe delivers 2 mL of 1:1 diluted Iopromide to confirm right renal artery opacification and save the images. (Figure 6A, Methods Video S7).

***Note:*** The diluent for iopromide is 0.9% sodium chloride solution.

**Figure 7:**
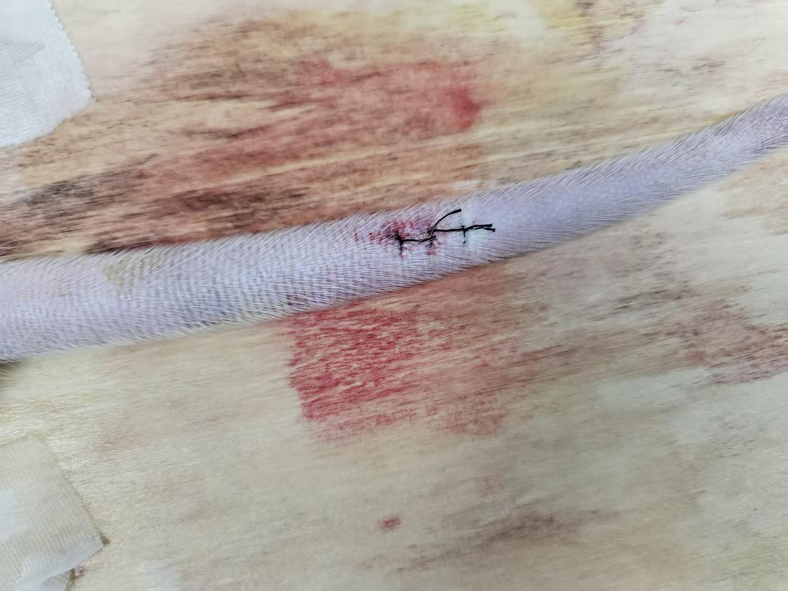
Suture of the rat caudal artery is completed

34. Inject 100 μL of microspheres through the microcatheter.
35. Reconnect the microcatheter to the high-pressure syringe and inject another 2 mL of 1:1 diluted Iopromide to confirm no opacification of the right renal artery. (Figure 6B and Methods Video S8).
36. After a 10-min wait, perform re-angiography (Figure 6C and Methods Video S9). If minimal recanalization of the right renal artery is observed, supplement with 50 μL of microspheres.
37. If the right renal artery fails to opacify, terminate the surgery.
38. Perform angiography again via the high-pressure syringe to observe the right renal artery (Figure 6D and Methods Video S10).
39. Terminate the angiography.
40. Remove the microcatheter and ligate the caudal artery with 5–0 suture (Figure 7).
41. Suture the tail wound (problem 5-troubleshooting).

Methods Video S6. Injection of contrast medium through a microcatheter for visualization of the right renal artery, related to step 31

Methods Video S7. Angiography of the right renal artery using a high-pressure syringe, related to step 33

Methods Video S8. Angiography of the right renal artery using a high-pressure syringe after the first microsphere embolization, related to step 35

Methods Video S9. Angiography of the right renal artery is performed again 10 min after embolization of the right renal artery, related to step 36

Methods Video S10. Angiography of the right renal artery after the second injection of embolization microspheres, related to step 38

## Expected outcomes

The primary aim of this experiment is to perform the puncture with minimal hemorrhagic risk. A 1.6F microcatheter is successfully introduced into the caudal artery through the sequential replacement of indwelling catheters of different specifications, followed by super-selective cannulation into the right renal artery for microsphere administration, ultimately achieving right renal artery embolization to validate the efficacy of detecting microsphere embolization (Figure 6D and Methods Video S10).

## Limitations

While our methodology demonstrates a certain degree of general applicability and currently achieves success in most rats, individual variability among rats may lead to situations where blood vessels are thinner or anatomical variations occur. In such cases, targeted preparation of corresponding specialized guidewires, catheters, and indwelling needles may be required to address these exceptional conditions.

## Troubleshooting

### Problem 1

Beginners may fail to master the force during tail incision, easily incising the caudal artery directly and causing massive hemorrhage (Related to Step 15).

### Potential solution

Beginners may consider making a lateral incision on the rat’s tail to avoid the caudal artery. However, this approach may more easily cause accidental injury to small tail veins, leading to local minor bleeding, and the dissection of the caudal artery may become more complicated.

### Problem 2

During arterial puncture, the artery may be pierced through or ruptured (Related to Step 18).

### Potential solution

This is a common issue encountered by operators during the procedure. When the puncture needle angle is too steep, it may pierce through the caudal artery; alternatively, excessive force applied when dissecting the caudal artery with a glass probe may rupture the artery. Both situations can complicate subsequent puncture. It is recommended that operators enlarge the surgical incision, continue dissecting the caudal artery toward the proximal end, and ask an assistant to retract the caudal artery posteriorly to provide tension for proximal puncture. If massive hemorrhage occurs or the caudal artery cannot be located, the experimental rat should be discarded, and a new rat used for the experiment.

### Problem 3

The microcatheter is prone to getting stuck when passing through the caudal artery into the abdominal aorta (Related to Step 28).

### Potential solution

**Figure 8:**
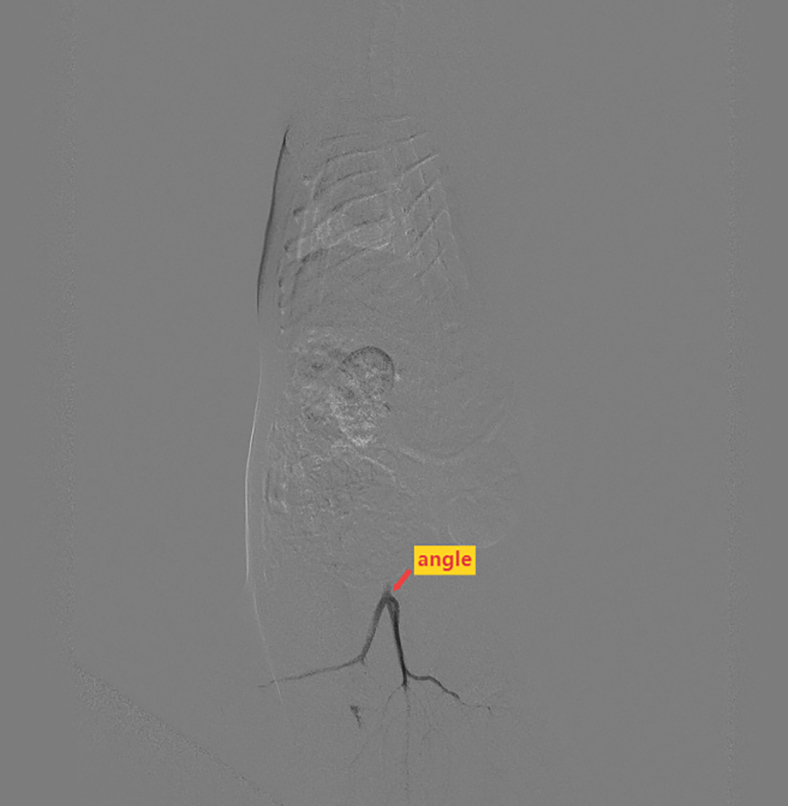
Withdrawing the microcatheter to the caudal artery for angiography reveals an angle at the entrance of the caudal artery into the abdominal aorta

The reason can be observed from the lateral angiography videos and images (Methods Video S11, Figure 8) of rats, where an angle exists at the junction of the caudal artery and abdominal aorta. If catheter sticking occurs, the anatomical position of the micro-guidewire can be visualized via lateral radiographs to guide it through the angle, thus enabling the microcatheter to smoothly enter the abdominal aorta.

Methods Video S11. Withdrawing the microcatheter to the root of the caudal artery, lateral angiography of the rat caudal artery and abdominal aorta reveals an angle where the caudal artery enters the abdominal aorta, related to problem 3

### Problem 4

During angiography of the right kidney, some terminal renal arteries may fail to opacify (Related to Step 32).

### Potential solution

The possible cause is that the micro-guidewire enters the renal artery too deeply, leading to spasm of some renal arteries. It is recommended to consider injecting a small amount of lidocaine then waiting for 15–20 min before performing angiography again to observe whether the renal arteries are fully open.

### Problem 5

The postoperative unexplained death of rats (Related to Step 41).

### Potential solution

There are multiple possible causes for postoperative death in rats, including excessive intraoperative bleeding, anesthesia overdose, postoperative infection, and excessive use of contrast agents during the operation. It is worth noting that for a rat weighing 300 g, the recommended volume of diluted Iopromide (1:1 dilution) should not exceed 9 mL. This recommended dosage is based on the clinical reference that the contrast agent used in 60 kg adults during surgery should not exceed 300 mL.

## Resource availability

### Lead contact

Further information and requests for resources should be directed to and will be fulfilled by the lead contact, Yi-nan Ding (dingyinanwl@126.com).

### Technical contact

Further information and requests for technical issues should be directed to and will be fulfilled by the technical contact, Yi Jiang (jiangyi@zjcc.org.cn).

### Materials availability

This study did not generate new unique reagents.

### Data and code availability

This study did not generate any unique datasets or code.

## Acknowledgments

The authors greatly appreciate the financial support from the 10.13039/501100002858China Postdoctoral Science Foundation (2023M743559 and 2024M763333), 10.13039/100014717National Natural Science Foundation of China (82472078 and 82202274), Zhejiang Medical and Health Science and Technology Program (2025KY674 and 2025KY686), and Zhejiang Province Natural Science Foundation (LTGD24H160009, LQN25H160008, and LTGC24B040001). We thank Dr. Jia-ping Zheng, Prof. Xu-feng Ni, and Prof. Jun Ling for help in establishing the protocol.

## Author contributions

Y.J., P.-t.C., C.-y.Q., and C.-h.S. helped with protocol optimization and wrote the manuscript. W.-j.W. and H.-y.D. participated in the design of the protocol and provided valuable suggestions. Y.-n.D. developed the protocol and wrote the manuscript.

## Declaration of interests

The authors declare no competing interests.
